# Supplementary material for: Assessment of endophytic bacterial diversity in rose by high-throughput sequencing analysis
Source: PLoS One. 2020 Apr 2;15(4):e0230924. doi: 10.1371/journal.pone.0230924 (PMC7117697; doi:10.1371/journal.pone.0230924)
Supplement: Attachment — Submitted filename: Response to Reviewers.doc [file pone.0230924.s001.doc]

Dear Reviewers,

Thank you for your letter and for the reviewers’ comments concerning our manuscript entitled “Assessment of endophytic bacterial diversity in Rose by high-throughput sequencing analysis” Those comments are all valuable and very helpful for revising and improving our paper, as well as the important guiding significance to our researches. We have studied comments carefully and have made correction which we hope meet with approval. Revised portion are marked in red in the paper. The main corrections in the paper and the responds to the reviewer’s comments are as flowing:

Reviewer #1:

1. The criterion for selecting the 18 rose varieties is that these 18 varieties have a large planting area and a wide planting area in China. Each rose is planted over 10hm^2^ at the sampling site.
2. These sequences have not been submitted to GenBank and can be submitted later if necessary.
3. High throughput sequencing (HTS) technologies, such as 454 pyrosequencing and Illumina sequencing, have been extensively developed and used to explore the microbial diversity of *Triticum aestivum* L, *Oryza sativa* L, fue-cured tobaccos, *Jasminum sambac* (L.) Ait, etc.
4. The gene expression analysis results were obtained by comparing the functional analysis of metagenomic sequencing data with the corresponding 16s predictive function analysis results.
5. I have added more details to comment on the future prospects of the present study in the manuscript.

In all, I found the reviewer’s comments are quite helpful, and I revised my paper point-by-point. Thank you and the review again for your help!

Reviewer #2:

1.I quite appreciate your favorite consideration and insightful comments and found these comments are very helpful. I have modified it according to your suggestion.

The 18 rose varieties have a large planting area and a wide planting area in China. Each rose is planted over 10hm^2^ at the sampling site. Plant tissue is rose petals. Each rose petal sample comes from more than 3 roses, and the growth distance exceeds 100 meters. Sample processing reference Wang et al. (2018), with some modifications.

The surface of the rose sample is sterilized by rinsing with sterile water, and the last rinsed water (0.01mL) were spread onto the Plate Count Agar (PCA) to check the sterilization effect. Samples with completely sterile surfaces were rinsed with distilled water more than three times to remove the remaining microbial DNA.

Rose petal samples have negative controls when amplifying bacterial DNA, and only the successfully amplified samples are sequenced.

Because the bacterial DNA content directly amplified is relatively low in rose petal samples, Because of the low content of bacterial DNA directly amplified in rose petal samples, the microbial diversity was analyzed by liquid culture and enrichment.

I describe more detail the quality control and data preparation information in the manuscript.

Regarding the venn diagram, I have modified it in the manuscript.

The functional gene predictions results were obtained by comparing the functional analysis of metagenomic sequencing data with the corresponding 16s predictive function analysis results. The accuracy of this method is 84% to 95%. The gene prediction is an inference in this article. The accuracy of 16S functional gene prediction can be referred to Langille et al. (2013). Thanks for your suggestion, I will more caution in the presentation and interpretation of these results.

2. I have revised the content of the results, discussion and conclusion.

3. If you need any more data, please don’t hesitate to contact me.

We appreciate for Reviewers’ warm work earnestly, and hope that the correction will meet with approval.

Once again, thank you very much for your comments and suggestions.

Yours sincerely,

Corresponding author:

Name: Yun-Guo Liu

E-mail: [yguoliu@163.com](mailto:yguoliu@163.com)

College of Life Sciences, Linyi University, Linyi 276005, China

Tel: 0086-539-7258711

Fax: 0086-539-725871

15 January 2020

Langille, M. G., Zaneveld, J., Caporaso, J. G., McDonald, D., Knights, D., Reyes, J. A., Huttenhower, C. (2013). Predictive functional profiling of microbial communities using 16S rRNA marker gene sequences. *Nat Biotechnol, 31*(9), 814-821. doi: 10.1038/nbt.2676

Wang, F., Men, X., Zhang, G., Liang, K., Xin, Y., Wang, J., Wu, L. (2018). Assessment of 16S rRNA gene primers for studying bacterial community structure and function of aging flue-cured tobaccos. *AMB Express, 8*(1), 182. doi: 10.1186/s13568-018-0713-1

Wu, Z., Hao, Z., Sun, Y., Guo, L., Huang, L., Zeng, Y., Chen, B. (2016). Comparison on the structure and function of the rhizosphere microbial community between healthy and root-rot Panax notoginseng. *Applied Soil Ecology, 107*, 99-107. doi: 10.1016/j.apsoil.2016.05.017
