# Supplementary material for: Assessment of endophytic bacterial diversity in rose by high-throughput sequencing analysis
Source: PLoS One. 2020 Apr 2;15(4):e0230924. doi: 10.1371/journal.pone.0230924 (PMC7117697; doi:10.1371/journal.pone.0230924)
Supplement: Attachment — Submitted filename: Comment_PONE.docx [file pone.0230924.s002.docx]

**# General Comment**

Although MS written well and illustrate the diversity; community composition of endophytes in native roses. The manuscript could be accepable for publication after the correction of following points.

1. MS should be check for plagiarism through authentic resources like turnitin or other acceptable software. This MS showed 22% plagiarism from different published resources like

i. Afusat Yinka Aregbe, Taihua Mu, Hongnan Sun. "Effect of different pretreatment on the microbial diversity of fermented potato revealed by high throughput sequencing" , Food Chemistry, 2019 (3% plagiarism from this paper).
ii. Amb-express.springeropen.com (3% plagiarism from this paper).
iii. Xinhui Wang, Songhu Wang, Hai Zhao. "Unraveling microbial community diversity and succession of Chinese Sichuan sausages during spontaneous fermentation by high throughput sequencing" , Journal of Food Science and Technology, 2019 (3% plagiarism from this paper).

Manuscript should be thoroughly revised. MS should not have match the same paragraph with other published article. Moreover, references mention in MS should be of original research articles.

2. References should be in journal format.
