# Supplementary material for: Assessment of endophytic bacterial diversity in rose by high-throughput sequencing analysis
Source: PLoS One. 2020 Apr 2;15(4):e0230924. doi: 10.1371/journal.pone.0230924 (PMC7117697; doi:10.1371/journal.pone.0230924)
Supplement: Attachment — Submitted filename: Response to Reviewers.doc [file pone.0230924.s003.doc]

Dear Reviewers,

Thank you for your letter and for the reviewers’ comments concerning our manuscript entitled “Assessment of endophytic bacterial diversity in Rose by high-throughput sequencing analysis” We have studied comments carefully and have made correction which we hope meet with approval. Revised portion are marked in red in the paper. The main corrections in the paper and the responds to the reviewer’s comments are as flowing:

Reviewer #3:

1. I have modified the content that may involve plagiarism.

2. The reference format has been modified.

We appreciate for Reviewers’ warm work earnestly, and hope that the correction will meet with approval.

Yours sincerely,

Corresponding author:

Name: Yun-Guo Liu

E-mail: [yguoliu@163.com](mailto:yguoliu@163.com)

College of Life Sciences, Linyi University, Linyi 276005, China

Tel: 0086-539-7258711

Fax: 0086-539-725871

9 February 2020
